# Correlation of apolipoprotein A‐I with T cell subsets and interferon‐ү in coronary artery disease

**DOI:** 10.1002/iid3.797

**Published:** 2023-03-14

**Authors:** Xinlin Xiong, Zonggang Duan, Haiyan Zhou, Guangwei Huang, Li Niu, Zhenhua Luo, Wei Li

**Affiliations:** ^1^ Department of cardiology The Affiliated Hospital of Guizhou Medical University Guiyang city Guizhou Province People's Republic of China; ^2^ Department of cardiology Clinical Medical College& Affiliated Hospital of Chengdu University Chengdu city Sichuan Province People's Republic of China; ^3^ Department of Central Lab, Department of Respiratory and Critical Care Medicine, Guizhou Provincial People's Hospital The Affiliated People's Hospital of Guizhou Medical University Guiyang city Guizhou Province People's Republic of China; ^4^ Guizhou University School of Medicine Guiyang city Guizhou Province People's Republic of China

**Keywords:** apolipoprotein A‐I, coronary artery disease, immune, inflammation, interferon‐ү

## Abstract

**Background:**

The association of Apolipoprotein A‐I (APOAI) with T cell subsets and interferon‐ү (IFN‐γ) in patients with coronary artery disease (CAD) has been not reported. Thus, this study aimed to investigate the association of APOAI with T cell subsets and IFN‐γ in CAD.

**Methods:**

This study included a total of 107 patients with CAD including acute coronary syndrome and chronic coronary syndrome. T cell subsets, and CD3‐CD56+ natural killer cells were quantified by flow cytometric analysis. The serum concentrations of IFN‐ү were measured by enzyme‐linked immunosorbent assay. Lipid profiles, C‐reactive protein (CRP), and fibrinogen were measured in the clinical laboratory. Clinical data was obtained duration hospitalization.

**Results:**

The CD4+ T cells were higher in patients of the low‐APOAI group (<median: 1.2 mmol/L) than in patients of the high‐APOAI group(≥median: 1.2 mmol/L) (*p* < .05). The CD8+ T cells were lower in patients of the low APOAI group than in patients of the high‐APOAI group (*p* < .05). APOAI was inversely associated with CD4+ T cells, IFN‐γ, and was positively associated with CD8+ T cells (*p* < .05). No correlation was observed between CD3 + CD56+ cells, regulatory T cells (Tregs), and CD3‐CD56+ natural killer cells and APOAI (*p* > .05). The high‐density lipoprotein cholesterol (HDL‐C) was also inversely associated with CD4+ T cells (*p* < .05), and positively associated with CD8+ T cells (*p* < .05). Lastly, APOA1 and HDL‐C did not correlated with fibrinogen and CRP (*p* > .05).

**Conclusion:**

The present study demonstrated the correlation of APOAI with T cell subsets and IFN‐γ in CAD. These results provided novel information for the regulatory action between APOAI and T cell subsets and inflammatory immunity in CAD.

## Graphical abstract

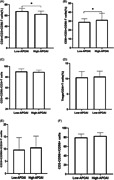

## 1 INTRODUCTION

Coronary artery disease (CAD) results from the comprehensive action of multiple factors such as diabetes mellitus, smoking, dyslipidemia, and obesity, and is linked to high morbidity and mortality worldwide.^1^, ^2^ Accumulating studies have demonstrated that atherosclerosis is an inflammatory immune cell‐ and lipid‐driven disease.^2^, ^3^ Immune response and inflammation are associated with CAD development, which is characterized by the formation of foam cells, infiltration of immune cells and cytokines, and lipid deposition.^3^ White blood cells could increase the risk for significant coronary stenosis, noncalcified plaques, and cardiovascular diseases.^4^

Moreover, dyslipidemia plays an important role in the initiation and development of atherosclerosis. Atherogenic lipids such as low‐density lipoprotein cholesterol (LDL‐C) are modified by oxidation and deposited in the sub‐endothelial area. Oxidized LDL can promote the expression of adhesion molecules on endothelial and smooth muscle cells, which can attract monocytes and lymphocytes into the vascular wall. Oxidized LDL activates T cells and macrophages. Finally, atherosclerotic plaques form, and these atherosclerotic lesions contain T cells, cytokines, accumulative lipids, and foam cells.^2^, ^3^, ^5^

Apolipoprotein A‐I (APOAI), the major structural and functional protein of high‐density lipoprotein‐cholesterol (HDL‐C), constitutes approximately 70% of HDL protein.^6^ APOAI could increase the cholesterol efflux from peripheral cells, and APOAI transfers peripheral cholesterol incorporated into HDL with the help of the ATP‐binding cassette transporter protein A1 from the peripheral tissues or cells to the liver, a process called reverse cholesterol transport.^6^ Thus, APOAI could regulate the cholesterol homeostasis in cells.

Increasing evidence have shown that APOAI or HDL has an immunomodulatory effect on innate and adaptive immune responses such as monocytes, macrophages, neutrophils, and T cells.^7^, ^8^, ^9^ These immunomodulatory roles have been elaborated in some diseases such as experimental arthritis,^10^, ^11^ colitis,^12^ systemic lupus erythematosus,^13^ graft‐versus‐host disease,^14^ autoimmune encephalomyelitis in mice models.^15^ Besides, in human studies, the associations of AOPAI or HDL with sepsis, acute pancreatitis, autoimmune disease, cancers have been reported.^16^, ^17^, ^18^, ^19^, ^20^ Manipulating APOAI has also been thought to be a novel treatment strategy for CAD. A study demonstrated that the use of APOAI mimetic peptides could improve atherosclerosis and plaque progression.^21^ A study of patients with hypertension also showed a negative association of HDL‐C level with leukocytes and lymphocytes.^22^ Another study revealed that HDL‐C was inversely associated with white blood cells in patients with CAD.^23^ However, to the best of our knowledge, the association of APOA1 or HDL‐C with T cell subsets and interferon‐ү (INF‐ү) in patients with CAD has been not reported. The correlation of APOA1 with T cell subsets and inflammatory makers in patients with CAD should be elucidated. Accordingly, this study investigated the association of APOAI with T cell subsets and IFN‐γ in CAD.

## 2 PATIENTS AND METHODS

### 2.1 Patients

This study included a total of 107 patients who had undergone coronary angiography at the Affiliated Hospital of Guizhou Medical University (from May 2021 to August 2022). The diagnostic criteria for CAD was based on the combination of ischemic clinical manifestations, electrocardiograms and troponin alteration, and coronary angiogram findings.^24^, ^25^, ^26^, ^27^, ^28^ The patients with CAD had at least one main coronary artery stenosis with a luminal diameter of ≥50%. CAD was composed of acute coronary syndrome (ACS) and chronic coronary syndrome (CCS). The median age of patients with CAD was 63.00 (55.00, 72.00) years. Patients with CAD who had either of the following medical histories were excluded: human immunodeficiency virus infection, malignancy, valvular heart disease, organ dysfunction (such as severe liver, or renal dysfunction), septicemia, and steroid therapy. This study was approved by the Ethics Committee of the Affiliated Hospital of Guizhou Medical University. Informed consent was obtained from all participants. This study was also in compliance with the ethical standards of Declaration of Helsinki.

### 2.2 Clinical data collection

Demographic data, such as age, sex, and medical history, and anthropometric parameters, such as body mass index (BMI), weight, and height, were obtained from the electronic medical system. Lipid profiles, C‐reactive protein (CRP), and fibrinogen were measured in the clinical laboratory.

### 2.3 Flow cytometric analysis of lymphocytes in the peripheral blood

Heparin‐anticoagulated whole blood was incubated with fluorophore‐conjugated antibodies against CD3, CD4, CD8, CD25, CD127, and CD56 at room temperature for 20 min. After erythrocytes were lysed using red blood cell lysate, the stained cells were washed once. The resuspended cells were analyzed by flow cytometry using the BD FACSCelesta flow cytometer equipped with Diva software. The CD3, CD4, CD8, CD25, and CD56 antibodies were purchased from BD Biosciences, and the antibody against CD127 was purchased from BioLegend.

### 2.4 Detection of IFN‐γ by enzyme‐linked immunosorbent assay (ELISA)

Blood samples were collected in a tube without anticoagulants, and the sera were separated, aliquoted, and stored at −80°C until analysis. The serum concentrations of IFN‐γ (Multiscience, China) were measured by ELISA based on the manufacturer's instructions.

### 2.5 Statistical analyses

Continuous variables were presented as mean ± standard deviation or medians with interquartile range. Categorical variables were presented as proportions. The *χ* ^2^ or Fisher's exact test was adopted for the comparisons of categorical variables. Comparison of continuous variables with normal distribution between two groups was carried out by Student's *t*‐test, or comparison of the nonparametric variables was carried out by Mann−Whitney *U* tests. The association of APOAI with T cell subsets, CD3‐CD56+ cells, fibrinogen, and IFN‐ү were analyzed by spearman's correlation coefficient. The multivariable line regression analysis was used to assess the association of T cell subsets, and CD3‐CD56+ cells with APOAI and HDL‐C levels. APOAI and HDL‐C were treated as independent variables and forcibly entered into the model, respectively. Other covariates were entered into the model with the stepwise method. All data were analyzed using IBM SPSS Statistics for Windows, version 26.0 (IBM Corp.). Values with a two‐sided *p* Value of <.05 were considered significant.

## 3 RESULTS

### 3.1 Baseline characteristics of patients with CAD according to APOAI median

These patients were stratified into low‐APOAI (<1.2 mmol/L) and high‐APOAI groups (≥1.2 mmol/L) according to APOAI median (1.2 mmol/L). The baseline characteristics of the patients are shown in Table 1. Patients in the low‐APOAI group had a higher prevalence of smoking, and higher frequency of male than those in the high‐APOAI group (*p* < .05). The HDL‐C and total cholesterol (TC) levels were decreased in low‐APOAI group, compared with high‐APOAI group. However, no differences were noted in the distribution of patients with ACS and CCS, diabetes mellitus, hypertension, statin use, triglyceride, age, and BMI between two groups.

**Table 1:** Baseline characteristic of patients according to apolipoprotein A‐I median.

Characteristics	Low‐apolipoprotein A‐I group (<1.2 mmol/L, *n* = 53)	High‐apolipoprotein A‐I group (≥1.2 mmol/L, *n* = 54)	*p*
Age (years)	61.00 (54.50−70.50)	66.50 (54.50−73.00)	NS
BMI (kg/m^2^)	25.04 ± 3.04	24.22 ± 3.21	NS
Male (%)	43 (81.10)	34 (63.00)	.036
Hypertension, *n* (%)	27 (50.90)	36 (66.70)	NS
diabete, *n* (%)	16 (30.20)	17 (31.50)	NS
smoking, *n* (%)	35 (66.00)	19 (35.20)	.001
ACS/CCS	35/18	37/17	NS
Statins, *n* (%)	15 (28.30)	13 (24.10)	NS
Total cholesterol	3.93 ± 1.15	4.44 ± 1.06	.020
Triglyceride	1.41 (0.84−2.40)	1.61 (1.04−2.63)	NS
HDL‐C	0.89 (0.76−1.00)	1.11 (1.02−1.35)	<.001

Abbreviations: ACS, acute coronary syndrome; BMI, body mass index; CCS, chronic coronary syndrome; HDL‐C, high density lipoprotein cholesterol; NS, not statistical significant.

### 3.2 Distribution of T cell subsets, and CD3‐CD56+ cells according to APOAI median

**Figure 1:**
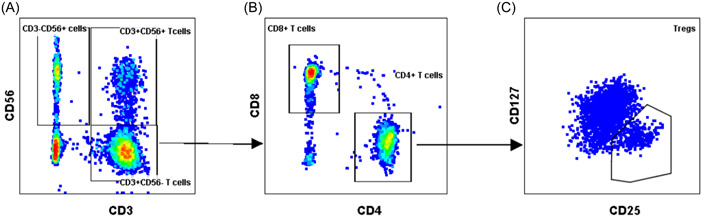
The gating strategy of the T cell, its subsets, and CD3‐CD56+ cells. (A) CD3/CD56 dot plot was used to identify CD3 + CD56‐cells, CD3‐CD56+ cells, and CD3 + CD56+ cells. (B) CD4/CD8 dot plot was used to identify CD4+ T cells and CD8+ T cells. (C) CD4 + CD25 + CD127^‐/low^ T cells(Tregs).

**Figure 2:**
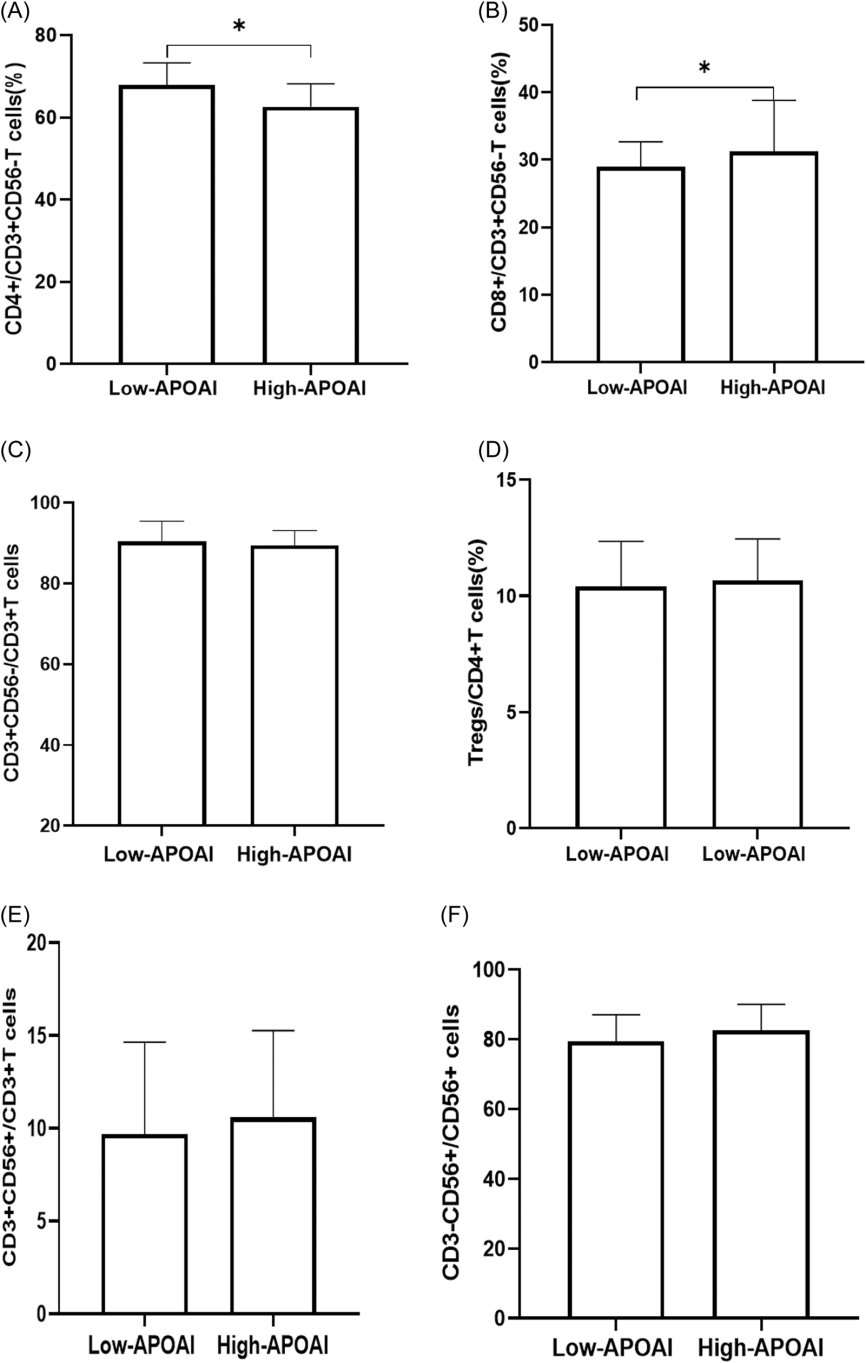
Differences in T cell subsets, and CD3‐CD56+ cells according to apolipoprotein A‐I median. (A) CD4+ T cells, (B) CD8+ T cells, (C) CD3 + CD56‐T cells, (D) CD4 + CD25 + CD127^‐/low^(Tregs), (E) CD3 + CD56+ T cells, (F) CD3‐CD56+ cells. (*Indicates *p* < .05). CD3+ T cells included CD3 + CD56‐T cells and CD3 + CD56+ T cells; CD56+ cells included CD3‐CD56+ cells and CD3 + CD56+ T cells.

The gating strategy of T cell subsets, and CD3‐CD56+ cells is presented in Figure 1. The number of CD4+ T cells was higher in the low‐APOAI group than in the high‐APOAI group (*p* < .05) (Figure 2A). The number of CD8+ T cells was lower in the low‐APOAI group than in the high‐APOAI group (Figure 2B). No difference was noted in CD3 + CD56+ T cells, CD3‐CD56+ cells, CD3 + CD56‐ T cells, and regulatory T cells (Tregs) between two groups (Figure 2C–F).

### 3.3 Spearman's correlation analysis of APOAI with T cell subsets, and CD3‐CD56+ cells

**Figure 3:**
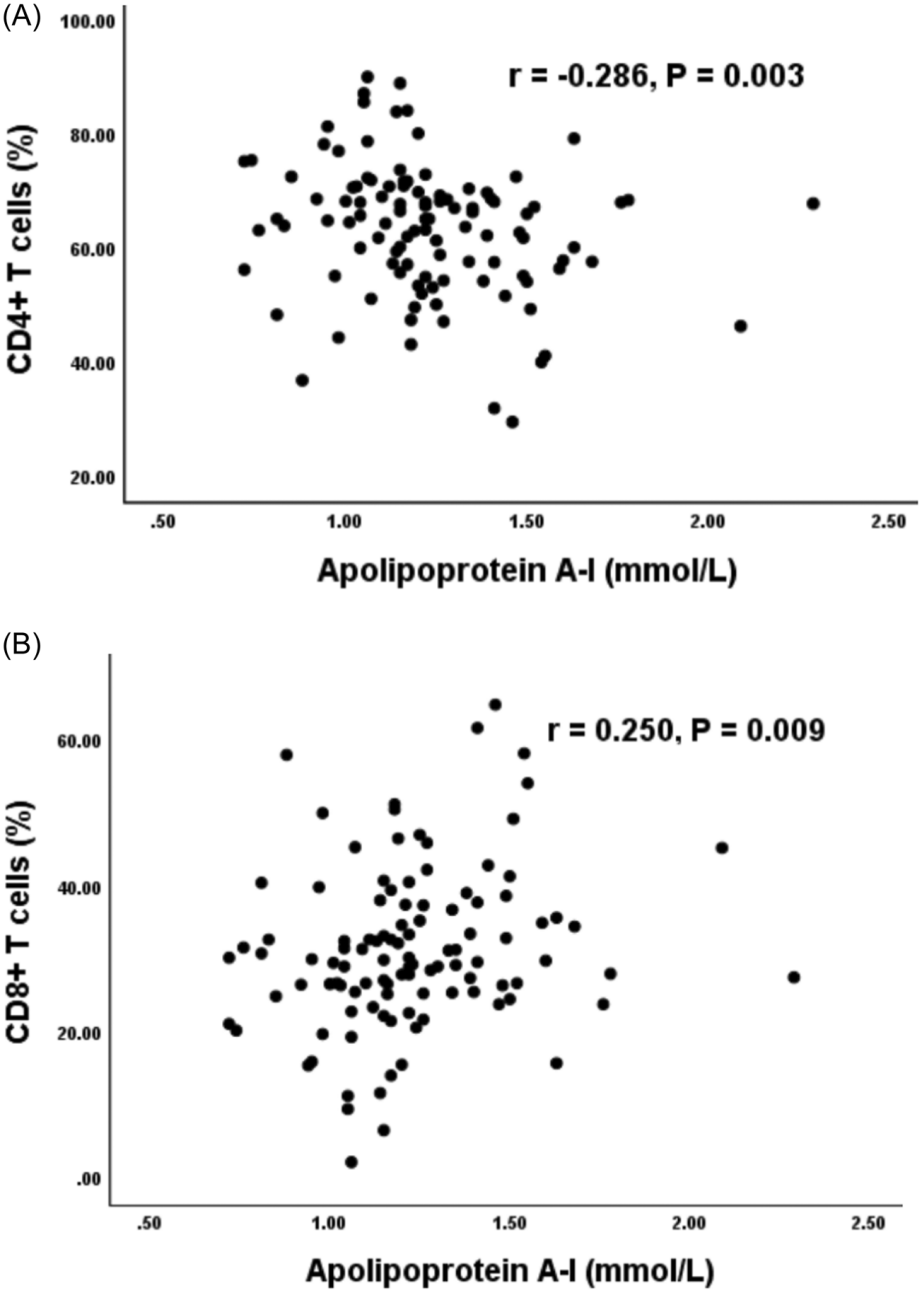
The association of Apolipoprotein A‐I with CD4+ and CD8+ T cells. Apolipoprotein A‐I negatively correlated with CD4+ T cells (A) and positively correlated with CD8+ T cells (B).

The APOAI was associated with CD4+ T cells (*r* = −0.286, *p* = .003, Table 2 and Figure 3A), and CD8+ T cells (*r* = 0.250, *p* = .009, Table 2 and Figure 3B) in patients with CAD. However, CD3‐CD56+ cells, CD3 + CD56− T cells, Tregs, and CD3 + CD56+ T cells did not correlate with APOAI (Table 2).

**Table 2:** Correlations between T cell subsets, CD3‐CD56+ cells and Apolipoprotein A‐I.

Variables	*r*	*P*
CD3 + CD56− T cells	−0.038	.699
CD4+ T cells	−0.286	.003
CD8+ T cells	0.250	.009
CD4 + CD25 + CD127^low/−^ cells(Tregs)	0.058	.550
CD3 + CD56+ cells	0.038	.699
CD3‐CD56+ cells	0.086	.379

*Note*: Correlation analysis was evaluated with spearman correlation.

### 3.4 The Independent correlations between APOAI and T cell subsets, and CD3‐CD56+ cells

We performed a multivariable stepwise regression analysis to assess the correlation of APOAI with T cell subsets, and CD3‐CD56+ cells. After taking into account these variables including age, sex, BMI, type of CAD (ACS and CCS), hypertension, diabetes mellitus, smoking, triglyceride (TG), TC, and statin use. APOAI was negatively related to CD4+ T cells (*β* = −.286, *p* = .005) and positively related to CD8+ T cells (*β* = .341, *p* = .001)(Table 3), whereas we found that APOAI was not associated with CD3‐CD56+ cells, CD3 + CD56+ T cells, CD3 + CD56− T cells, and Tregs (Table 3).

**Table 3:** The correlations between T cell subsets, CD3‐CD56+ cells and apolipoprotein A‐I.

Dependent variables	Other variables included in the models	Standardization coefficient *β* with apolipoprotein A‐I as independent variables	*p*
CD3 + CD56− T cells	Covariates	0.030	.762
CD4+ T cells	Covariates	−0.286	.005
CD8+ T cells	Covariates	0.341	.001
CD4 + CD25 + CD127^low/−^ cells (Tregs)	Covariates	0.071	.470
CD3 + CD56+ T cells	Covariates	−0.030	.762
CD3‐CD56+ cells	Covariates	0.026	.786

*Note*: Multivariable stepwise regression analyses were performed to identify correlation of apolipoprotein A‐I with T cell subsets, and CD3‐CD56+ cells in different models. Apolipoprotein A‐I was independent variable. Covariates included age, sex, body mass index (BMI), hypertension, smoking, diabetes mellitus, triglyceride, total cholesterol, type of coronary artery disease, statins use.

### 3.5 The Independent correlations between HDL‐C with T cell subsets, and CD3‐CD56+ cells

**Figure 4:**
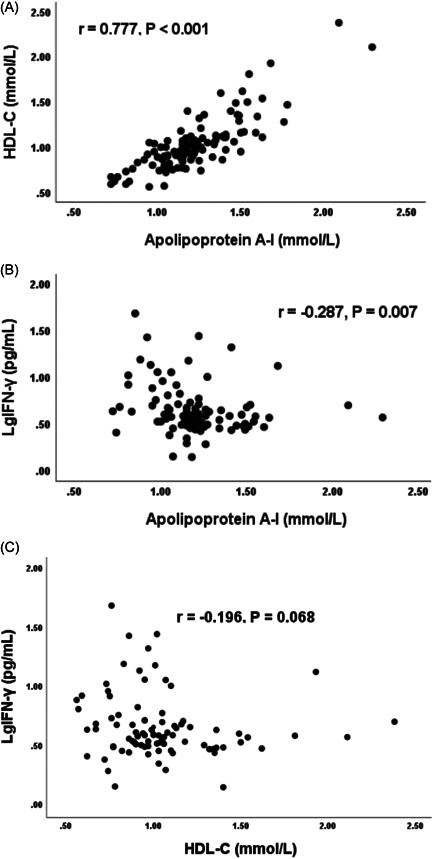
The association of Apolipoprotein A‐I with high density lipoprotein cholesterol (HDL‐C), and Interferon‐ү (IFN‐ү). Apolipoprotein A‐I positively correlated with HDL‐C (A). Apolipoprotein A‐I negatively correlated with IFN‐ү (B). HDL‐C did not correlate with IFN‐ү (C). IFN‐ү values were logarithmically transformed.

HDL‐C consists mainly of APOAI,^6^ and HDL‐C is closely related to APOAI (*r* = 0.777, *p* < .001, Figure 4A). Thus, we also assessed the correlation of HDL‐C with T cell subsets, and CD3‐CD56+ cells, which will further prove the correlation of APOAI with T cell subsets, and CD3‐CD56+ cells from the perspectives of HDL‐C. We performed multiple line stepwise regression analysis after controlling for age, sex, BMI, hypertension, diabetes mellitus, smoking, CAD type including ACS and CCS, TG, TC, and statin use. The HDL‐C was found to be inversely associated with CD4+ T cells (*β* = −.224, *p* = .024), and positively related to CD8+ T cells (*β* = .259, *p* = .010). However, a significant association of CD3‐CD56+ cells, CD3 + CD56+ T cells, CD3 + CD56− T cells, and Tregs with HDL‐C was also not observed (Table 4).

**Table 4:** The correlation of T cell subsets, CD3‐CD56+ cells with HDL‐C.

Dependent variables	Other variables included in the models	Standardization coefficient *β* with HDL‐C as independent variables	*p*
CD3 + CD56− T cells	Covariates	0.030	.764
CD4+ T cells	Covariates	−0.224	.024
CD8+ T cells	Covariates	0.259	.010
CD4 + CD25 + CD127^low/‐^ cells(Tregs)	Covariates	0.145	.139
CD3 + CD56+ cells	Covariates	−0.030	.764
CD3‐CD56+ cells	Covariates	0.073	.451

*Note*: Multivariable stepwise regression analysis were performed to identify correlation of high‐density lipoprotein cholesterol(HDL‐C) with T cell subsets, and CD3‐CD56 + cells in different models. HDL‐C was independent variable. Covariates included age, sex, body mass index(BMI), hypertension, smoking,diabetes mellitus, triglyceride, total cholesterol, type of coronary artery disease, statins use.

### 3.6 Correlation of makers of inflammation with APOAI

We examined the association of APOAI with IFN‐γ, CRP, and fibrinogen. IFN‐γ data of 88 patients were available. Spearman's correlation analysis showed that APOAI correlated with IFN‐γ (*r* = −0.287, *p* < .05, Figure 4B). CRP data of 55 patients were available. APOAI did not correlate with CRP (*p* > .05). Additionally, HDL‐C was not associated with IFN‐γ (Figure 4C) and CRP (*p* > .05). Lastly, APOA1 and HDL‐C did not correlated with fibrinogen (*p* > .05).

## 4 DISCUSSION

To the best of our knowledge, this is the first study to find the correlation of APOAI with T cell subsets and IFN‐ү in patients with CAD. The major finding of this study was that the APOAI and HDL‐C levels were inversely and independently correlated with CD4+ T cells, and positively and independently correlated with CD8+ T cells in patients with CAD. APOAI levels were aslo negatively associated with IFN‐γ. Furthermore, we did not find the association of APOAI and HDL‐C with CD3‐CD56+ cells, CD3 + CD56+ T cells, CD3 + CD56− T cells, Tregs, and fibrinogen. Nevertheless, this study may provide new insights concerning the association of APOAI with immune inflammation in CAD.

T cells, marked by CD3, include CD4+ T cells, CD8+ T cells, and CD3 + CD56+ T cells. Moreover, CD4+ T cells were classified into CD4+ T helper cells (Th) such as Th1, Th2, Th17, and Tregs.^5^ Numerous studies have shown that T cells and its subsets were associated with CAD or atherosclerosis. T cells including CD4+ T cells and CD8+ T cells were found to be increased in atherosclerotic lesions.^5^, ^29^ An earlier study reported that T cells were found in plaques of human carotid artery specimens.^30^ Another study reported that CD4+ T cells were major proatherogenic cells in a mice model of atherosclerosis.^31^ The transfer of CD4+ T cells from apoE−/− mice into immunodeficient apoE−/− mice could promote the progression of atherosclerotic lesions.^32^ The absence of CD4+ T cells in apoE‐knockout mice could ameliorate atherosclerosis.^31^ A study revealed that CD8+ T cells played an obvious role in advanced atherosclerotic plaques.^33^ CD8+ T cells accounted for approximately 50% of the lymphocytes in advanced lesions in human arterial tissue.^33^ The depletion of CD8+ T cells in apo E‐deficient mouse model improved atherosclerosis.^34^ The above findings indicated that T cells play important roles in the pathogenesis of CAD and atherosclerosis.

An animal study indicated that APOAI could inhibit the proliferation of CD4+ memory T cell subsets (CD4 + CD44^high^ T cells) in the lymph nodes in the LDLr −/–, APOAI −/− (DKO) mouse model.^35^ Another study reported that APOAI deficiency could expand T cells in the mouse model.^36^ A study showed that synthetic peptides of APOAI could inhibit allogeneic T‐lymphocyte proliferation in vitro.^37^ The above studies have suggested that APOAI inversely regulated T cells.

Several studies have reported the correlation of APOAI or HDL‐C with T cell subsets. Zhao et al. reported that HDL‐C was inversely associated with CD3+ T cells and CD4+ T cells in elderly patients with type 2 diabetes mellitus (T2DM).^38^ Another study reported that HDL‐C was inversely associated with CD4+ T subsets including interleukin (IL)‐8‐expressing CD4+ T cells and IL‐17‐expressing CD4+ T cells in diabetes mellitus patients with CAD. However, the association was not observed among these participants with T2DM, T2DM without CAD, and healthy controls.^39^ Moreover, Guasti et al. found that HDL and APOAI were not associated with CD4+ T cells in patients with dyslipidemia and healthy controls.^40^ In present study, we found a negative and independent correlation of APOAI or HDL‐C with CD4+ T cells in CAD. Based on the results of our study and those of the aforementioned studies, we speculated that APOAI might negatively regulate CD4+ T cells in CAD. The interaction of APOAI with T cells may be involved in the pathogenesis of CAD or atherosclerosis.

Tregs play important roles in atherosclerosis. It could be found in atherosclerotic plaques. Multiple studies have shown that Tregs helped to control the progression of atherosclerotic lesions.^41^, ^42^, ^43^ Although several studies have assessed the correlation of HDL‐C or APOAI with Tregs in different patients including those with diabetes, dyslipidemia, and healthy individuals, the results remain controversial.^40^, ^44^, ^45^, ^46^ Studies have shown that FOXP3+ regulatory T cells were positively associated with HDL‐C in healthy participants and in participants with T2DM.^44^, ^45^ Others reported contrary results. Wigren et al. reported that they did not find a significant association of Tregs with lipid profiles including HDL‐C, LDL‐C, and TG in a prospective cohort study.^46^ In present study, we found no association of APOAI or HDL‐C with Tregs in CAD. Our findings were in agreement with the study by Wigren et al.^46^ The difference in the results of various studies is probably related to the differences in the study participants, and diseases state.

A previous literature reported that APOAI had anti‐atherosclerotic action by some functions such as reverse cholesterol transport, inhibiting the expression of cell adhesion molecules, depleting cholesterol of lipid rafts, and exerting antioxidative effects.^9^, ^47^ Using ATP binding cassette transporters A1 and G1 (Abca1/g1)‐deficient mice, Westerterp M et al. found that reduced cholesterol efflux in dendritic cells could lead to their activation and production of inflammatory cytokine.^48^ Cholesterol accumulation in antigen‐presenting cells could promote T cell priming, APOAI treatment improved reverse cholesterol transport and the immune disorder.^49^ Our findings, in combination with those of previous studies,^23^, ^35^, ^36^, ^37^ indicated that the regulation of APOAI to CD4+ T cells may be another key step and mechanism for anti‐atherosclerosis in CAD. We speculated that the negative correlation of APOAI with CD4+ T cells is caused by APOAI or HDL‐C's inhibition of the ability of antigen‐presenting cells to present antigen to T cells, influencing cholesterol efflux, lipid raft disruption or production of some inflammatory cytokines.^10^, ^37^, ^47^, ^48^, ^49^, ^50^, ^51^

Interestingly, we found that APOAI and HDL‐C were positively correlated with CD8+ T cells, because human specimens and animal experiments revealed that CD8+ T cells contributed to the development of atherosclerosis.^33^, ^34^ This appeared to be contrary to the anti‐atherosclerotic role of APOAI and HDL‐C. An earlier study showed that HDL‐C was negatively correlated with the risk of CAD,^52^ whereas other studies have reported that the upregulation of HDL‐C level did not lower the risk of CAD.^53^, ^54^ One of the possible reasons for this result is the positive regulatory role of APOAI and HDL‐C with CD8+ T cells. However, the precise mechanisms warrant further investigation in different diseases, especially CAD.

IFN‐γ has important proatherogenic effects. IFN‐γ is produced by various cells including CD4+ T cells and CD8+ cells. In mouse models, exogenous IFN‐γ administration increases the atherosclerotic lesions.^55^ By contrast, the loss of IFN‐γ signaling can reduce atherosclerotic lesions in ApoE−/− mice and LDLR−/− mice.^56^, ^57^ In patients with CAD, the evidence from the study showed that IFN‐γ was obviously increased in the CAD group compared with the control group and was associated with the occurrence of CAD. ^58^, ^59^

IFN‐γ has also been characterized as a functional and hallmark cytokine of Th1 cells and can induce Th1 differentiation. In human atherosclerotic lesions, Th1 cells are predominant effector cells, which are associated with the progression of the lesion.^60^ Moreover, IFN‐γ modulates other inflammatory cells including monocytes, macrophages, and foam cells to take part in and promote atherosclerosis.^61^ IFN‐γ exposure can lead to endothelial dysfunction of human coronary artery endothelial cells.^62^

HDL and APOAI could enable the inhibition of inflammation. They could prohibit the secretion of chemokines such as CCL2 and CX3CL1, reduce their receptor expression, and adhesion molecule expression on endothelial cells.^6^ Besides, APOAI suppressed proinflammatory cytokines secreted by monocytes by blocking the interaction between monocytes and stimulated T cells.^63^ A study reported that HDL treatment inhibit IL‐6, and tumor necrosis factor‐α, and IL‐4 production by Mycobacterium tuberculosis‐infected macrophages.^64^ IFN‐γsecretion was suppressed by HDL or APOAI treatment.^10^, ^37^, ^64^ Moreover, APOA1 or HDL reduced the production of IFN‐ү by CD8+ T cells.^14^ However, previous studies have not reported the association of APOAI or HDL‐C with IFN‐γ in patients with CAD. In present study, we found that APOAI was also inversely related to the IFN‐γ level, together with the above studies, suggesting that APOAI may exert anti‐inflammatory response, at least in part, by negatively regulating the production of IFN‐γ in CAD. However, the association between HDL‐C and IFN‐γ was not observed, which was inconsistent with the correlation between APOAI and IFN‐γ. The inconsistent result may be attributed to that HDL included various ingredients such as apolipoprotein A‐II, apolipoprotein E,^6^ which could interfere with the association of HDL‐C with IFN‐γ. Moreover, CRP was thought to be an inflammatory marker, as it has been reported in some inflammatory diseases including CAD. We also assessed the association of APOAI and HDL‐C with CRP, whereas we did not find a significant correlation of APOAI or HDL‐C with CRP. These findings, in combination with those of previous studies, indicated that the regulatory correlation of APOAI and HDL‐C with T cell subsets, and IFN‐γ may play an important role in the pathogenesis of CAD. Fibrinogen was not only inflammatory representation but also coagulation markers.^65^ In present study, however, a significant correlation of AOPAI and HDL‐C with Fibronogen was not observed.

This study has some limitations. First, T cells could be further divided into subtypes such as IFN‐ү‐secreting CD4+ T cells (Th1), Th2, Th17, and IFN‐ү‐secreting CD8+ T cells. The correlation of APOAI with these subtypes of T cells deserves much further exploration. Second, we analyzed regulatory CD4+ T (Treg) cells based on the low expression of CD127 and the expression of CD25, but not directly using the intracellular expression of FoxP3. Even if this is a widely approach used to identify Treg, the prototypical marker of Treg is the transcription factor, FoxP3. Thirdly, we have only analyzed a subset of Treg, the association of APOAI with other Treg cells such as IL‐10‐secreting Treg cells (Tr1) cells remains to be further studied. Moreover, in patients with CAD, besides IFN‐ү, many other cytokines are abnormally secreted by immune cells, whether APOAI also could inhibit these cytokines remains to be further studied. Finally, we did not obtain the medication duration and dosage of statins use, which may limit further correlation analysis.

## 5 CONCLUSIONS

In conclusion, we firstly revealed that APOAI was negatively correlated with CD4+ T cells, and IFN‐γ, and positively correlated with CD8+ T cells in CAD. Together with the findings of previous studies, the present results provided novel important information for the modulatory action and anti‐inflammatory response between APOAI and T cell subsets, and inflammatory markers in CAD. However, the exact signal pathways and molecular mechanisms between APOAI and T cells and inflammatory markers require further investigation in patients with CAD or atherosclerosis. Our ongoing studies will elucidate these issues.

## AUTHOR CONTRIBUTIONS

**Xinlin Xiong**: Conceptualization; data curation; formal analysis; investigation; methodology; project administration; software; validation; writing—original draft; writing—review and editing. **Zonggang Duan**: Conceptualization; formal analysis; methodology; software. **Haiyan Zhou**: Conceptualization; formal analysis; methodology; software; validation; writing—original draft; writing—review and editing. **Guangwei Huang**: Formal analysis; methodology; software. Li Niu: formal analysis; investigation; methodology; software. **Zhenhua Luo**: Conceptualization; formal analysis; funding acquisition; methodology; project administration; resources; supervision; validation; writing—original draft; writing—review and editing. **Wei Li**: Conceptualization; data curation; formal analysis; funding acquisition; investigation; methodology; project administration; resources; supervision; validation; visualization; writing—original draft; writing—review and editing.

## CONFLICT OF INTEREST STATEMENT

The authors declare no conflict of interest.

## ETHICS STATEMENT

This study was approved by the Ethics Committee of the Affiliated Hospital of Guizhou Medical University (approval number: 2021010K).

## Data Availability

The data that support the findings of this study are available from the corresponding author, upon reasonable request.
